# William Frederick Rehfuss, D.D.S.

**Published:** 1893-05

**Authors:** 


### WILLIAM FREDERICK REHFUSS, D.D.S.

Died, of Bright's disease, on the 28th of March, Dr. William F. Rehfuss, aged twenty-six years.

Dr. Rehfuss was born in Philadelphia, March 1, 1867, of German parentage. He received his education in the public schools, graduating with honor at the Central High School in 1884.

He began his professional studies with Dr. Thomas Robson, of Philadelphia, with whom he remained one year. He matriculated in the Department of Dentistry, University of Pennsylvania, graduating in 1887.

During his student life he was the representative from this department on the editorial staff of the college paper, *The Pennsylvanian.*

After graduating he opened an office in Philadelphia, and also one during the summer months at Ocean Grove, N. J.

He was an original worker, spending his leisure hours principally in his private laboratory writing and experimenting.

The fact that no book on dental jurisprudence existed, early claimed his attention, and with his usual energy he proceeded to carefully train himself to prepare such a work. He labored unceasingly at this for several years. Very few, it is surmised, as they refer to this book now, can appreciate the great labor bestowed upon it,—a labor which undoubtedly laid the foundation of the disease which ended his career. When it was finally prepared, he was met by the discouraging fact that none of the large publishers would touch it. They naturally feared financial loss. To the credit of the Wilmington Dental Manufacturing Company, the risk was taken; and not only this, but they spared no expense to produce it in a satisfactory manner. The result justified the hopes of the author and their expectations, as it is now the standard work on the subject.

In addition to this labor, he has been a prolific contributor to various societies. His articles on dental massage were widely read, and regarded as of special value in a new line of treatment in dental pathological conditions. He also wrote on other topics. Among the few of the many written now recalled are "Antisepsis in Dentistry," "Abstract or Dental Laws," "Internal Medication in regard to Dentistry." The latest work on which he was engaged during his last sickness was a serial contribution, in collaboration with L. Brinkmann, M.D., on "Oral Diseases, Surgical and Non-Surgical,"

illustrated by colored plates. These articles were to be continued through the year.

He was a member of the Odontological Society of Pennsylvania, and at the time of his death its corresponding secretary. He was also a member of the New Jersey State Dental Society and the Dental Protective Association of the United States.

His interest in society-work and ability in the preparation of interesting papers led to frequent invitations to take part in distant organizations.

Socially he was well known in his native city, and during his earlier years took an active interest in amateur theatricals, having managed several of the leading amateur societies.

The trustees of the New York Dental School elected him Professor of Dental Jurisprudence, and he was looking forward to this work with great interest.

He was engaged to be married, at the time of his death, to Miss Helen Lawson, of Brooklyn, N. Y.

His funeral took place from his father's residence on March 31, 1893.

